# FSH induces EMT in ovarian cancer via ALKBH5-regulated Snail m6A demethylation

**DOI:** 10.7150/thno.94161

**Published:** 2024-03-03

**Authors:** Xingyan Xu, Xuefen Zhuang, Haowei Yu, Ping Li, Xiaosa Li, Huiping Lin, Jian-peng Teoh, Yiwen Chen, Yuanlan Yang, Yang Cheng, Weiyu Chen, Xiaodong Fu

**Affiliations:** 1 Guangzhou Institute of Cardiovascular Disease, the Second Affiliated Hospital, School of Basic Medical Sciences, Guangzhou Medical University, Guangzhou, China.; 2 Department of Gynecology and Obstetrics, The Sixth Affiliated Hospital, Guangzhou Medical University, Guangzhou, China.; 3 Department of Gynecology and Obstetrics, Guangzhou First People's Hospital, Guangzhou, China.

**Keywords:** Follicle-stimulating hormone, Epithelial ovarian cancer, Epithelial-mesenchymal transition, Snail, ALKBH5

## Abstract

**Background:** The therapeutic benefits of targeting follicle-stimulating hormone (FSH) receptor in treatment of ovarian cancer are significant, whereas the role of FSH in ovarian cancer progresses and the underlying mechanism remains to be developed.

**Methods:** Tissue microarray of human ovarian cancer, tumor xenograft mouse model, and *in vitro* cell culture were used to investigate the role of FSH in ovarian carcinogenesis. siRNA, lentivirus and inhibitors were used to trigger the inactivation of genes, and plasmids were used to increase transcription of genes. Specifically, pathological characteristic was assessed by histology and immunohistochemistry (IHC), while signaling pathway was studied using western blot, quantitative RT-PCR, and immunofluorescence.

**Results:** Histology and IHC of human normal ovarian and tumor tissue confirmed the association between FSH and Snail in ovarian cancer metastasis. Moreover, in epithelial ovarian cancer cells and xenograft mice, FSH was showed to promote epithelial mesenchymal transition (EMT) progress and metastasis of ovarian cancer via prolonging the half-life of *Snail* mRNA in a N6-methyladenine methylation (m6A) dependent manner, which was mechanistically through the CREB/ALKBH5 signaling pathway.

**Conclusions:** These findings indicated that FSH induces EMT progression and ovarian cancer metastasis via CREB/ALKBH5/Snail pathway. Thus, this study provided new insight into the therapeutic strategy of ovarian cancer patients with high level of FSH.

## Introduction

Ovarian cancer is most lethal gynecological malignancies 1, however, its pathogenic mechanism is remained to be elucidated 2. Theories of ovarian cancer pathogenesis, proposed that extended exposure to high levels of gonadotropin 3, *e.g.,* follicle-stimulating hormone (FSH), may excessively stimulate ovarian epithelium growth, ultimately contributing to tumor development 4, 5. These theories were supported by the observation that FSH was increased in peritoneal fluid of ovarian cancer patients 6. Studies have demonstrated FSH promoted proliferation 7, migration, and invasion 8 of epithelial ovarian cancer cell via FSH receptor (FSHR) 9. Antagonist of gonadotropin 10 and antibodies targeting surface-expressed FSHR 11-13 were shown the potential role of FSHR in ovarian cancer therapy, evident by suppression of tumorigenesis in mouse model of ovarian cancer. Nevertheless, in post-menopausal women undergoing hormone replacement therapy, a reduction in FSH was correlated with an elevated risk of ovarian cancer 14. Unexpectedly, study showed that FSH inhibited proliferation in normal ovarian epithelial cells from both premenopausal and postmenopausal women 15, which did not support the mentioned theories that gonadotrophin is directly involved in ovarian carcinogenesis. These contradictions underscore the critical need of investigation on the role of FSH in ovarian cancer and its underlying mechanisms.

The major cause of mortality in ovarian cancer patient is tumor metastasis 16. In metastasis, cells go through epithelial-mesenchymal transition (EMT), which involves epithelial cells losing their original surface markers and gaining characteristics of mesenchymal cells, thereby allowing tumors to detach from their original location and spreading to distant areas. EMT is collectively regulated by various transcription factors, *e.g.,* Snail, Slug, Twist, ZEB1, and ZEB2 17. Recent studies reported that EMT and its transcription factors were modulated by N6-methyladenosine (m6A) modifications 18-20, and intervention in m6A modifications demonstrated a potential cancer therapy 21.

mRNA m6A modification, a prevalent post-transcriptional modification, is dynamically regulated by methyltransferases (*e.g.,* METTL3, METTL14 and WTAP), demethylases (*e.g.,* ALKBH5 and FTO), and readers (*e.g.,* YTHDF and YTHDC family) 22. In the context of ovarian cancer, m6A plays a crucial role in mRNA synthesis, transcription, and degradation of oncogenes and tumor suppressor genes 23-25, ultimately influencing various tumorigenic processes, including cancer cell proliferation, migration, metastasis 26, chemoresistance 27 and immune evasion 28. The modification of mRNA by m6A in EMT transcription factors also impacts EMT and tumor metastasis 29-31. However, there is currently no clear evidence on whether FSH can regulate m6A modification on EMT transcription factors and influence ovarian cancer metastasis.

In this study, we observed that FSH induced the upregulation of the m6A demethylase ALKBH5 and led to the promotion of EMT in ovarian cancer cells. Further investigation revealed that ALKBH5 regulated the transcription factor Snail, a known suppressor of E-cadherin. Additionally, we uncovered that FSH upregulated ALKBH5 expression through cAMP-response element binding protein (CREB), resulting in the removal of m6A modifications on *Snail* mRNA. This process enhanced Snail expression and contributed to the initiation of EMT. The crucial role of ALKBH5 in FSH-induced ovarian cancer metastasis was confirmed in both animal model and clinical samples. Our findings suggested that intervening m6A modifications to modulate Snail could be a potential therapeutic approach for ovarian cancer.

## Results

### FSH induced EMT in epithelial ovarian cancer cells via demethylase ALKBH5

**Figure 1:**
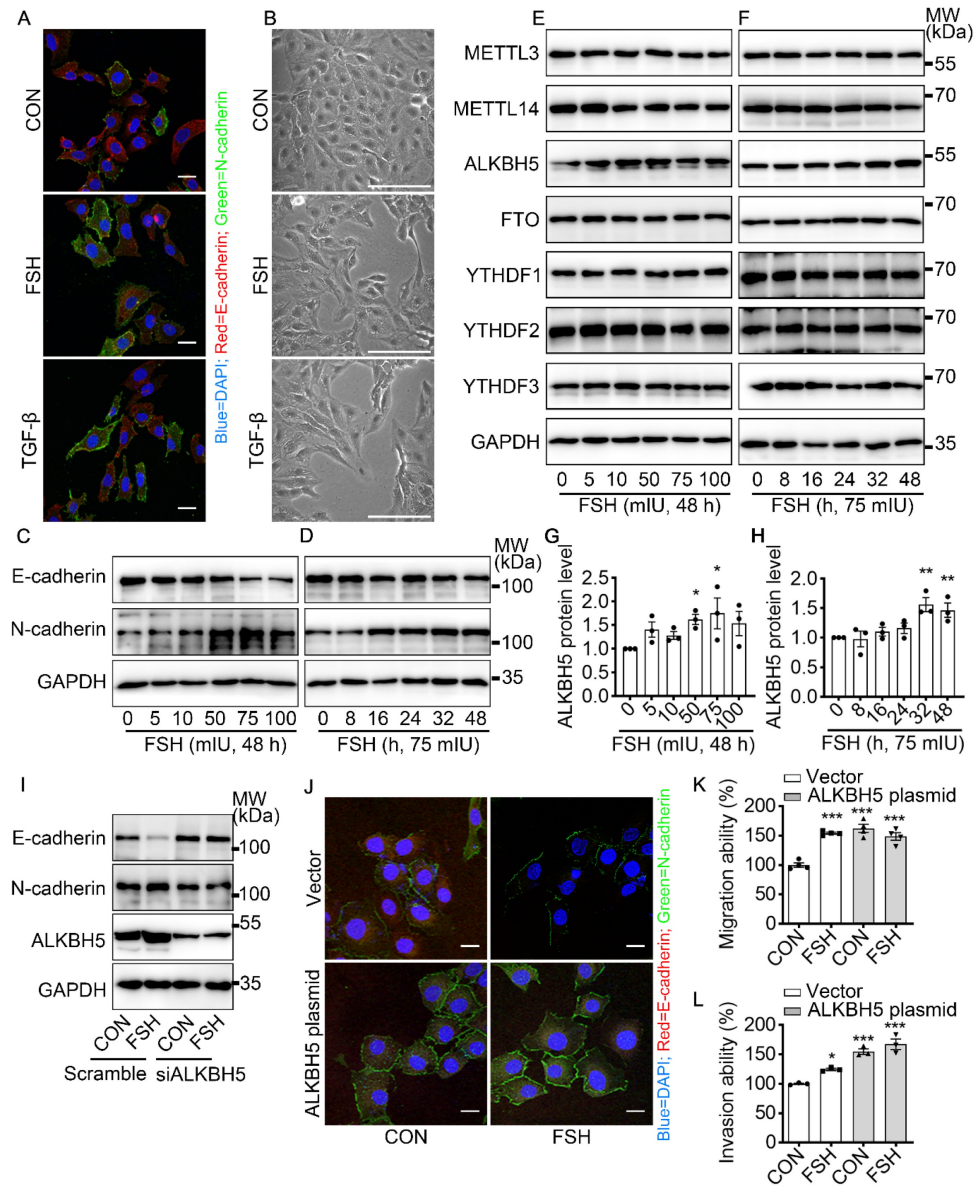
FSH induced EMT in epithelial ovarian cancer cells via demethylase ALKBH5. **(A, B)** Immunofluorescence and bright filed images of SK-OV3 cells treated with FSH (75 mIU) or TGF-β (10 ng/μl) for 48 hours, scale bar = 20 µm. **(C-H)** SK-OV3 cells were treated with different concentrations of FSH for various time points. **(C, D)** Western blots for E-cadherin and N-cadherin. **(E, F)** Western blots for METTL3, METTL14, ALKBH5, FTO and YTHDF1/2/3. **(G, H)** Western blot quantification of ALKBH5 intensity measurements (*n* = 3), data was analyzed by Mann-Whitney U test. *P < 0.05, **P < 0.01 *versus* CON.** (I)** Western blots of E-cadherin, N-cadherin and ALKBH5 in SK-OV3 cells treated with FSH (75 mIU, 48 h) after transfection of ALKBH5 siRNA (siALKBH5). **(J-L)** SK-OV3 cells transfected with ALKBH5 plasmid followed by FSH treatment (75 mIU, 48 h), *n* = 3 - 4 per group. (**J**) Immunofluorescence of E-cadherin- and N-cadherin-stained SK-OV3 cells. Scale bar = 20 µm.** (K, L)** Wound healing and Transwell assays were performed in SK-OV3 cells, data was analyzed by one-way ANOVA test. *P < 0.05, **P < 0.01, ***P < 0.001 *versus* CON transfected with Vector.

To get a broad view of the influence of FSH on the ovarian cancer, immunohistochemistry (IHC) of FSH receptor (FSHR) was performed on tissue microarray (TMA) of ovarian tumors derived from 160 ovarian cancer patients. As shown in Table 1 and Figure S1A, high expression of FSHR had a higher probability of metastasis. TGF-β has been determined to be a major inducer of EMT during cancer metastasis 32. Our result showed that FSH induced an EMT phenotype comparable to TGF-β in epithelial ovarian cancer cells (Figure 1A, B and Figure S1B). Moreover, the changes in E-cadherin and N-cadherin expression confirmed FSH-induced EMT in multiple epithelial ovarian cancer cell lines (Figure 1C, D and Figure S1C-E). Among the cell lines, SK-OV3 showed an obvious response and was then chose as a major *in vitro* tool to investigate the role of FSH in EMT.

Emerging studies have shown that m6A modification plays an essential role in various cancer progressions 33. To determine whether m6A modification was involved in FSH-induced EMT progression, we first investigated the expression patterns of seven key regulators for m6A modification, our results show that both demethylase ALKBH5 and methyltransferase METTL14 respond to FSH in epithelial ovarian cancer cells (Figure 1E-H and Figure S1F, H, I). Through a systematic analysis of the expression patterns of EMT and m6A regulators, Yanna Zhang *et al*. revealed a synergistic effect between ALKBH5 and EMT regulators on ovarian cancer development 34. Our results confirmed that ALKBH5 had higher expression in epithelial ovarian cancer cell lines (*e.g.,* SK-OV3, ES-2, and A2780) compared with the normal ovary cell line (*e.g.,* ISOE-80) (Figure S1G). The silence of ALKBH5 by siRNA inhibited the regulation of FSH on E-cadherin and N-cadherin expressions (Figure 1I, J and Figure S1J), while overexpression of ALKBH5 enhanced the regulation of FSH on E-cadherin and N-cadherin expressions, thereby reducing EMT-related activity in SK-OV3 and A2780 cells (Figure 1K, L and Figure S1K-M). Taken together, our result showed that demethylase ALKBH5 is essential for FSH-induced EMT progression in epithelial ovarian cancer cells.

### Snail is a critical transcription factor in FSH-induced EMT

**Figure 2:**
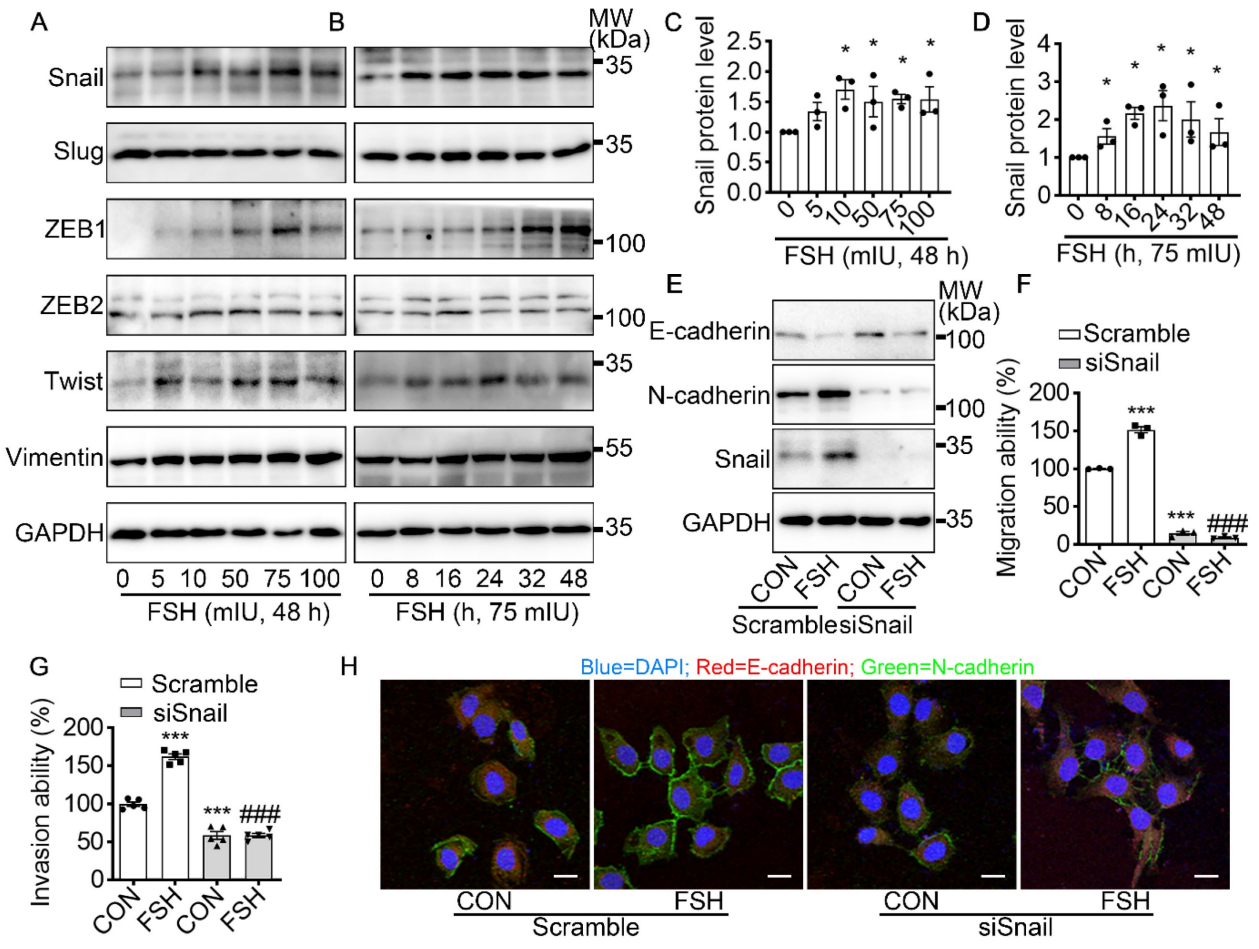
Snail is a critical transcription factor in FSH-induced EMT. **(A-D)** SK-OV3 cells were treated with different concentrations of FSH for various time points.** (A, B)** Western blots for Snail, Slug, ZEB1/2, Twist and Vimentin. **(C, D)** Western blot quantification of Snail intensity measurements (*n* = 3), data was analyzed by Mann-Whitney U test. *P < 0.05 *versus* CON.** (E-H)** SK-OV3 cells were treated with FSH (75 mIU, 48 h) after transfection of scramble or Snail siRNA (siSnail). **(E)** Western blots of E-cadherin, N-cadherin and Snail in SK-OV3 cells. **(F, G)** Wound healing and Transwell assays were performed. Quantitative data of healing area (*n* = 3) and cell invasion (*n* = 5) were analyzed by one-way ANOVA test. ***P < 0.001 *versus* CON group transfected with scramble siRNA, ###*P* < 0.001 *versus* FSH group transfected with scramble siRNA. **(H)** Immunofluorescence of SK-OV3 cells stained with E-cadherin and N-cadherin. Scale bar = 20 µm.

To further explore the underlying mechanism of FSH-induced EMT, we monitored the expression pattern of six main EMT-related protein after FSH treatment. Our result demonstrated that both EMT transcription factors (Snail, ZEB1, Twist) and EMT protein markers (Vimentin) were responds to FSH in epithelial ovarian cancer cells (Figure 2A-D and Figure S2A-D). Snail, a key inducer of EMT, have been reported to be involved in the chemoresistance 35, stemness, and metastasis 36 in ovarian cancer. To confirm the role of Snail in FSH-induced EMT, Snail siRNA was transfected to knock down Snail expression. The regulatory effects of FSH on E- and N-cadherin were abolished by knocking down Snail, indicating that FSH-induced EMT was dependent on Snail signaling (Figure 2E, H and Figure S2E, F). Similarly, FSH-induced migration and invasion of SK-OV3 were attenuated by Snail silencing (Figure 2F, G and Figure S2G, H). These results indicated that FSH promotes EMT of epithelial ovarian cancer cells via Snail signaling.

### m6A modification of *Snail* mRNA is essential for FSH-induced EMT via ALKBH5

**Figure 3:**
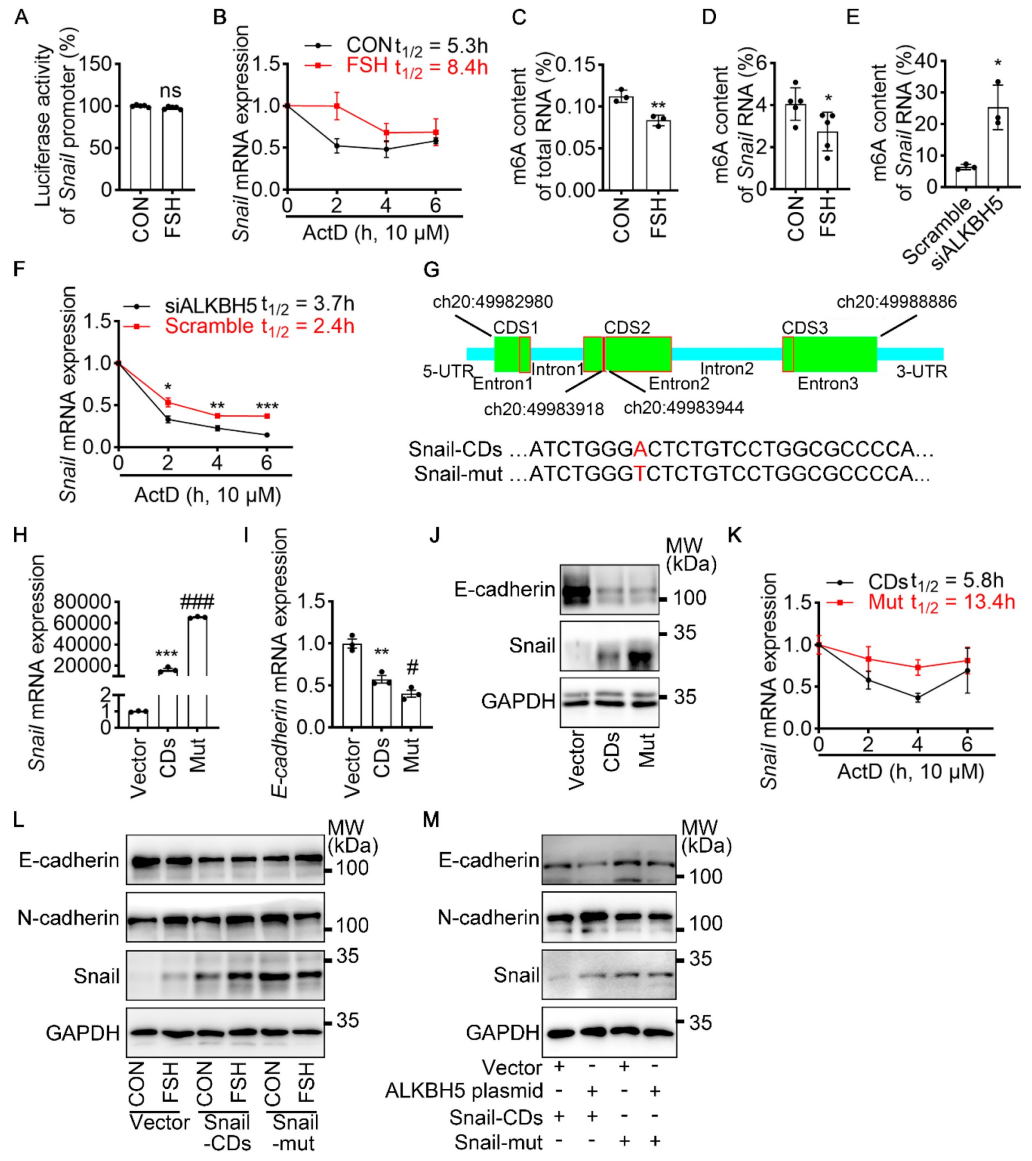
m6A modification of *Snail* mRNA is essential for FSH-induced EMT via ALKBH5. **(A)** Snail promoter activity was measured by luciferase assay in 293T cells transfected with FSHR plasmid before PBS or FSH (75 mIU, 48 h) treatment (*n* = 5), data was analyzed for statistical difference by two-tailed unpaired t test, ns, no significant. **(B)** qRT-PCR of *Snail* mRNA in SK-OV3 cells with pretreatment of FSH (75 mIU, 48 h) and treatment of Actinomycin D (ActD) for various time points (*n* = 3). **(C)** N6-methyladenosine (m6A) content of total RNA in SK-OV3 cells with FSH treatment (75 mIU, 48 h), *n* = 3 per group, data was analyzed by two-tailed unpaired t test. **P < 0.01. **(D)** m6A RIP-qPCR analysis of *Snail* mRNA in SK-OV3 cells treated with FSH (75 mIU, 48 h), *n* = 5 per group, data was analyzed by two-tailed unpaired t test. *P < 0.05. **(E)** m6A RIP-qPCR analysis of *Snail* mRNA in SK-OV3 cells transfected with ALKBH5 siRNA (siALKBH5) (*n* = 3), data was analyzed by two-tailed unpaired t test. *P < 0.05. **(F)** qRT-PCR of *Snail* mRNA in SK-OV3 cells pretreated with Actinomycin D (ActD) for various time points (*n* = 7), data was analyzed by two-tailed unpaired t test, *P < 0.05, **P < 0.01, ***P < 0.001. **(G)** Scheme showed locations of the m6A motifs within the *Snail* mRNA, and A to T mutation within m6A consensus site in *Snail* mRNA. **(H-K)** SK-OV3 cells were transfected with empty vector (Vector), Snail-CDs (CDs) and Snail-mut (Mut) plasmid. Data was analyzed by two-tailed unpaired t test, **P < 0.01, ***P < 0.001. *versus* Vector. #*P* < 0.05, ###*P* < 0.001 *versus* CDs.** (H)** qRT-PCR of *Snail* mRNA. **(I)** qRT-PCR of *E-cadherin* mRNA.** (J)** Western blots for E-cadherin and Snail. **(K)** RT-PCR of *Snail* mRNA in SK-OV3 cells transfected with Snail-CDs (CDs) and Snail-mut (Mut) plasmid and treatment of Actinomycin D (ActD) for various time points (*n* = 3). **(L)** Western blots of E-cadherin, N-cadherin, and Snail in SK-OV3 cells treated with FSH (75 mIU, 48 h), after transfection of Snail-CDs or Snail-mut plasmid.** (M)** Western blots for E-cadherin, N-cadherin and Snail in SK-OV3 cells co-transfected with ALKBH5 plasmid and wild type Snail (Snail-CDs) or CDS region mutated (Snail-mut) plasmid.

To revealed the specific role of Snail in FSH-induced EMT via ALKBH5, we first preformed luciferase assay for *Snail* promoter activity. However, we observed that FSH was not able to increase the transcriptional activity of *Snail* (Figure 3A). We then used actinomycin D to evaluate the stability of *Snail* mRNA. Result showed that FSH prolonged the half-life of *Snail* mRNA (Figure 3B). Studies had shown that m6A modification can extend the half-life of mRNA 37-39, therefore we determined whether m6A modification participate in increased mRNA and protein expression of Snail induced by FSH. Indeed, FSH was able to decrease m6A content of total RNA in SK-OV3 cells as indicated by methylation quantification assay (Figure 3C). According to the substrates preference of ALKBH5 40, sequence analysis was performed to predict Snail containing consensus m6A motifs located in the CDS2 region (Figure 3G). m6A-Immunoprecipitation (MeRIP) qPCR result confirmed that FSH reduces m6A modification of *Snail* mRNA in the predicted regions (Figure 3D). Sequentially, we confirmed ALKBH5 reduced both the mRNA expression and m6A modification level of *Snail* (Figure 3E and Figure S3A). Furthermore, we found ALKBH5 prolonged the half-life of *Snail* mRNA (Figure 3F). Our result also showed that ALKBH5 prolong Snail protein half-life using protein biosynthesis inhibitor cycloheximide (CHX) pulse-chase assay (Figure S3C, D). The role of ubiquitination in ALKBH5 regulated Snail expression was also assessed via using proteasome inhibitor (MG132), result showed progression of ubiquitination had limited influence on ALKBH5 regulated Snail expression (Figure S3B).

To further confirm the role of m6A modification on *Snail* mRNA in FSH-regulated EMT, a wild-type Snail (Snail-CDs) or mutant (Snail-mut) plasmid was constructed and transfected into SK-OV3. Both Snail-mut and Snail-CDs plasmid transfection decreased *E-cadherin* mRNA and protein level compared with vector. Compared with Snail-CDs plasmid transfection, an evaluated Snail expression was observed in Snail-mut plasmid transfection, interestingly, this increased Snail expression induced by the Snail-mut was not able to further decrease the expression of E-cadherin (Figure 3H-J, Figure S3E). Additionally, actinomycin D assay was performed and showed that m6A modification played an essential role in the stability of *Snail* mRNA (Figure 3K). Further result showed that FSH-induced EMT was blocked with by mutation of Snail on the m6A modification site (Figure 3L). We next assessed whether FSH reduces m6A modification of *Snail* mRNA via demethylase ALKBH5. Snail-CDS or Snail-mut was co-transfected with ALKBH5 plasmid in SK-OV3 cells. We found a significant decrease in E-cadherin and increase in N-cadherin, while co-transfection of Snail-mut and ALKBH5 plasmid was not able to recapitulate this phenomenon (Figure 3M).

To determine the essential role of ALKBH5 in the FSH-induced EMT process, ALKBH5 siRNA was solely- or co-transfected with Snail plasmid into SK-OV3 cells. We observed that knockdown of ALKBH5 inhibited FSH-promoted EMT, migration and invasion, while these inhibitory effects were rescued by the overexpression of Snail (Figure S3F-H). Thus, we confirmed that ALKBH5 was implicated in FSH-induced EMT by regulating Snail in a m6A-dependent manner. ALKBH5 H204A (catalytic inactive mutation) plasmid transfection 41 further demonstrated that the demethylase activity of ALKBH5 was essential in EMT (Figure S3I).

### FSH increases ALKBH5/Snail expression and triggers EMT via CREB

**Figure 4:**
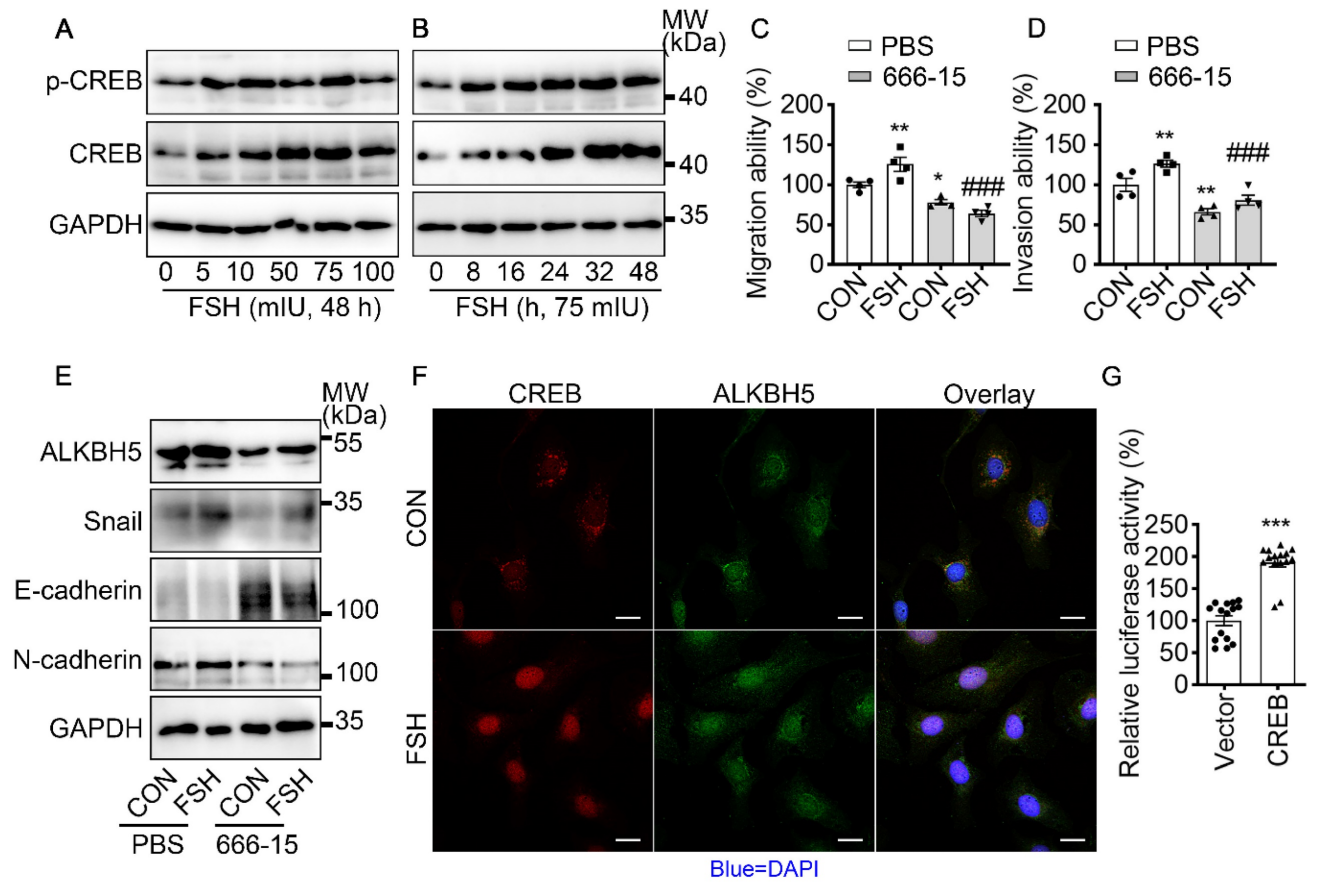
FSH activates CREB to increase ALKBH5 expression. **(A, B)** Western blots of p-CREB and CREB in SK-OV3 cells treated with different concentrations of FSH for various time points. **(C, D)** Wound healing and invasion assays were performed on SK-OV3 cells with pretreatment of CREB inhibitor 666-15 (10 μM, 1 h), then with FSH treatment (75 mIU, 48 h), *n* = 4 per group, data was analyzed for statistical difference by one-way ANOVA test. *P < 0.05, **P < 0.01* versus* CON+PBS, ###*P* < 0.001* versus* FSH+PBS. **(E)** Western blots of ALKBH5, Snail, E-cadherin and N-cadherin expression in SK-OV3 cells with pretreatment of 666-15 (10 μM, 1 h) and treatment of FSH.** (F)** Immunofluorescence for CREB and ALKBH5 in SK-OV3 cells treated with FSH (75 mIU, 48 h). Scale bar = 20 µm. **(G)** ALKBH5 promoter activity was measured by luciferase assay in SK-OV3 cells transfected with Vector or CREB plasmid (*n* = 15), data was analyzed by two-tailed unpaired t test. ***P < 0.001.

It is well established that cAMP response element-binding protein (CREB) is a classical transcription factor that can be phosphorylated and activated by FSH. To determine if CREB is involved in FSH-induced EMT, we first confirmed FSH activated CREB phosphorylation in a time- and concentration-dependent manner (Figure 4A, B and Figure S4A, B, I). Phosphorylated CREB acted as the transcription factor to regulate various gene transcription 42. To explore whether *ALKBH5* gene transcription was regulated by CERB, a selective inhibitor of CERB, 666-15, was used to inhibit CREB-mediated gene transcription 43. Our result showed FSH activates CREB to increase ALKBH5 and Snail expression, thereby enhancing migration and invasion of ovarian cancer (Figure 4C-E and Figure S4C-H, J). Moreover, bioinformatics analysis (http://jaspar.genereg.net) predicted that CREB binding site locates in *ALKBH5* gene promoter region (Figure S4K). Luciferase assay further confirmed that transcriptional activity of *ALKBH5* was regulated by CREB (Figure 4G). Moreover, we monitored that FSH induced accumulation of CREB and ALKBH5 in nucleus (Figure 4F). These findings indicated that CREB-triggered ALKBH5 gene transcription plays an important role in FSH-induced EMT and tumor metastasis.

### FSH enhances EMT and metastasis of ovarian cancer via ALKBH5 in model of xenografts

**Figure 5:**
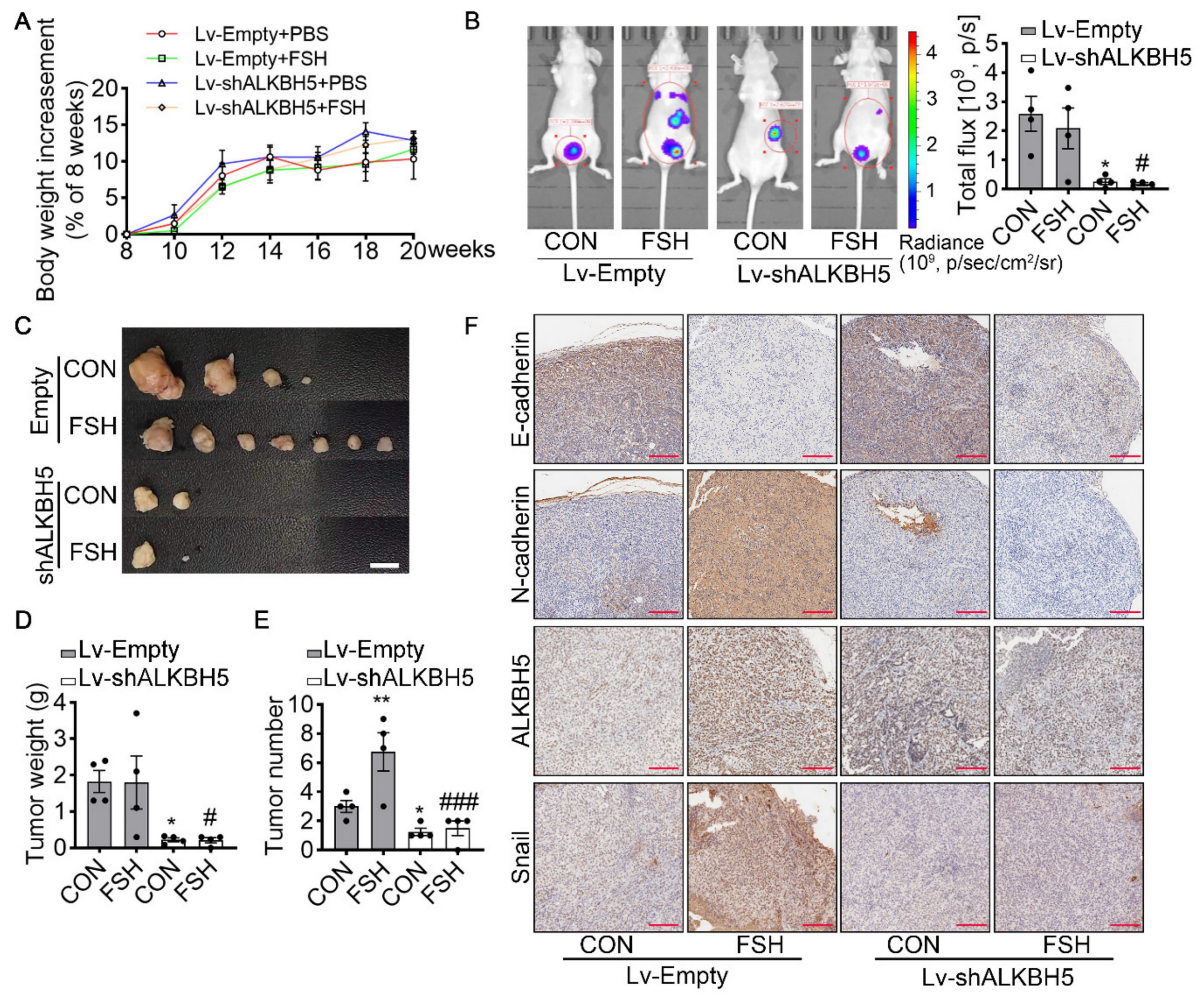
ALKBH5 is essential for FSH-induced EMT and metastasis of ovarian cancer in mouse xenografts model. (**A-F**) SK-OV3 cells were transfected with a control (Lv-Empty) or shALKBH5 (Lv-shALKBH5) luciferase-tagged lentivirus. SK-OV3 cells were then implanted to bilateral ovariectomized (OVX) nude mice to develop a xenografts model for FSH treatment (3 IU/day per mouse for 14 weeks). **(A)** Body increasement was calculated from the body weight of 8 weeks (*n* = 4). **(B)** *In vivo* bioluminescence imaging was performed to monitor tumor burden, and the bioluminescent signal was quantified and analyzed by Mann-Whitney U test (*n* = 4). *P < 0.05 *versus* CON group with Lv-Empty transfection. #*P* < 0.05 *versus* FSH group with Lv-Empty transfection.** (C-E)** Tumors of the SK-OV3 tumor-bearing mice were excised, imaged and weighed (*n* = 4). Scale bar = 1 cm. Data was analyzed by Mann-Whitney U test. *P < 0.05, **P < 0.01 *versus* CON group with Lv-Empty transfection. #*P* < 0.05, ###*P* < 0.001 *versus* FSH group with Lv-Empty transfection.** (F)** SK-OV3 xenografts were sectioned and stained for E-cadherin, N-cadherin, ALKBH5 and Snail expression by immunohistochemistry. Scale bar = 200 µm.

To verify the effect of ALKBH5 in FSH-promoted progression of epithelial ovarian cancer *in vivo*, the ovariectomized nude mice were intraperitoneally injected with SK-OV3 cells transfected with empty lentivirus (Lv-Empty) or ALKBH5 shRNA lentivirus (Lv-shALKBH5) to establish peritoneal metastatic models of ovarian cancer (Figure S5A). One week after the development of xenograft, mice were treated with FSH for 14 weeks (Figure S5B). During the 14 weeks treatment, there was no significant different in body weight among the 4 groups (Figure 5A). At the endpoint of experiment, *in vivo* imaging system (IVIS) was performed to assess the presence of peritoneal metastases. As expected, FSH increased the risk of distant metastasis and number of tumors (Figure 5B). However, ALKBH5 knockdown significantly blocked the effect of FSH on the risk of distant metastasis and tumor number in xenograft mice (Figure 5C-E). IHC result showed that FSH increased expression of N-cadherin, ALKBH5, Snail and decreased expression of E-cadherin in tumor tissues, while these effects were reversed by the knockdown of ALKBH5 (Figure 5F). Ki-67 protein has been widely used as a proliferation marker for human tumor cells. IHC of Ki-67 was performed and result showed both FSH treatment and Lv-ALKBH5 transfection had comparable influence on the proliferation level in SK-OV3 xenografts (Figure S5C). Taken together, our finding indicated that FSH increases ALKBH5 to enhance EMT and metastasis of ovarian cancer.

### Both FSHR and Snail expression were positively associated with ALKBH5 expression in ovarian cancer

**Figure 6:**
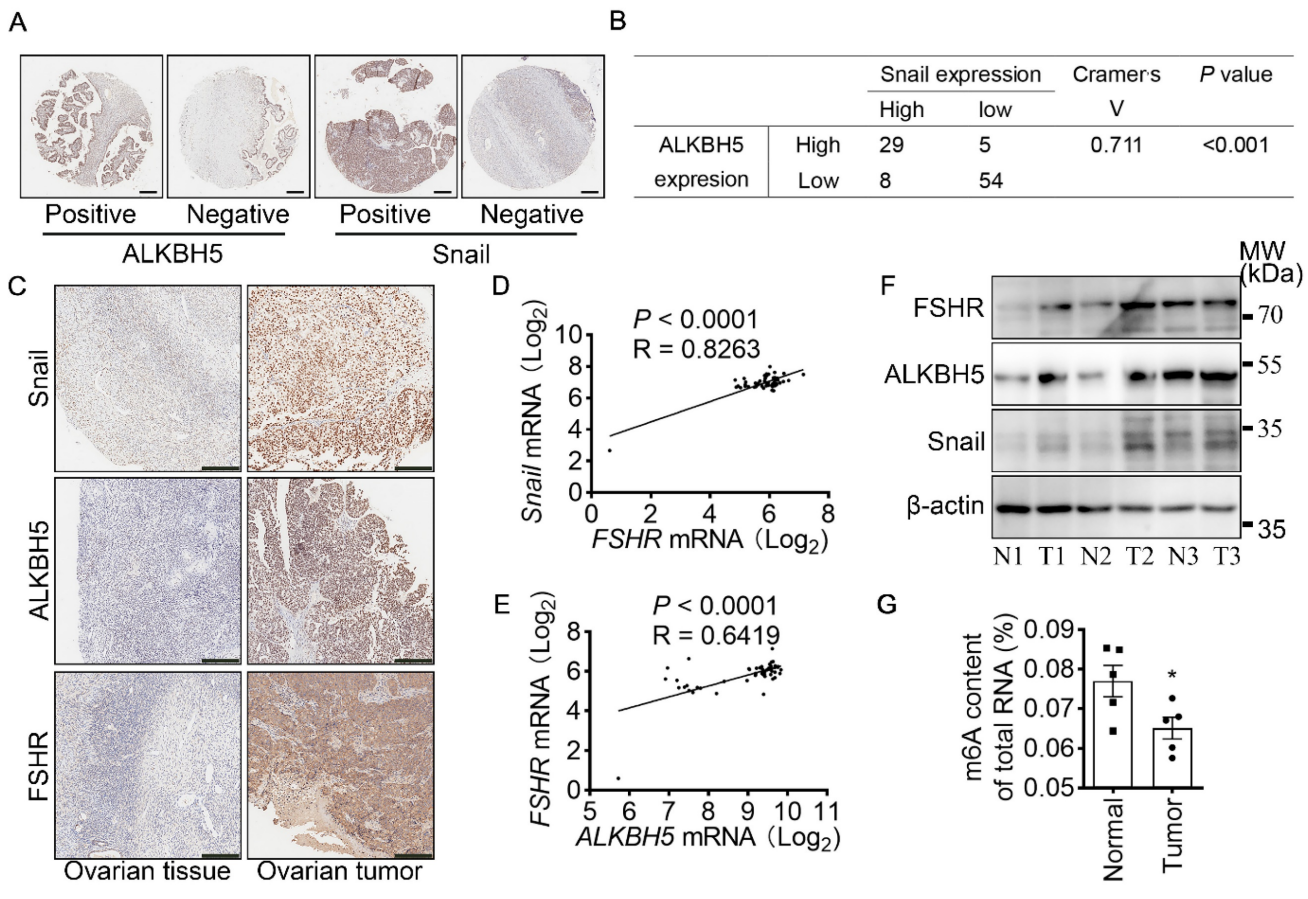
FSHR and Snail expression are positively associated with ALKBH5 expression. **(A)** IHC of ALKBH5 and Snail on tissue microarray of ovarian tumor. Scale bar = 250 μm. **(B)** Correlation of ALKBH5 to Snail expression in ovarian tumor. *Cramér's V* was used for the association analysis. *V* = 0.711, *P* < 0.001. **(C)** IHC of Snail, ALKBH5 and FSHR in tissue sections of normal and tumor ovarian tissues. Scale bar = 200 μm. **(D)** Association between *Snail* and *FSHR* mRNA expression in The Cancer Genome Atlas Program (TCGA, https://tcga-data.nci.nih.gov/tcga/tcgaHome2.jsp). *Cramér's V* was used for the association analysis. **(E)** Association between *ALKBH5* and *FSHR* mRNA expression in TCGA database. *Cramér's V* was used for the association analysis. **(F)** Western blots of FSHR, ALKBH5 and Snail protein in normal ovarian tissues (N) and ovarian tumors (T). **(G)** m6A content of total RNA in normal ovarian tissue and ovarian tumor (*n* = 5), data was analyzed by two-tailed unpaired t test. *P < 0.05.

To determine the role of ALKBH5 and Snail expression in clinicopathologic characteristics of ovarian cancer, IHC of ALKBH5 and Snail were performed on two adjacent TMA containing 88 ovarian tumors and 8 normal ovarian tissues of 96 individual patients (Table 2 and Figure 6A). Based on low and high expression of ALKBH5, patients were divided into two groups. As shown in Table 2, high expression of ALKBH5 was positively associated with ovarian cancer stages (*P* = 0.019), node metastasis (*P* = 0.04), distant metastasis (*P* < 0.001), peritoneal seeding (*P* = 0.001), ascites formation (*P* < 0.001), primary tumor origin (*P* = 0.04), histological type (*P* = 0.037). By performing IHC of Snail and ALKBH5 on the TMA, we further determined the correlation between Snail and ALKBH5 expression using *Cramér's V* association analysis (*V* = 0.711, *P* < 0.001, Figure 6B). As shown in Table 2, high expression of Snail was correlated to patients age (*P* = 0.019), ovarian cancer stages (*P* < 0.001), distant metastasis (*P* < 0.001), peritoneal seeding (*P* < 0.001), ascites formation (*P* < 0.001), primary tumor origin (*P* = 0.038).

To confirm the role of FSHR, ALKBH5 and Snail in the progression of ovarian cancer, expressions of FSHR, ALKBH5 and Snail in paired freshly normal ovarian (from hysteromyoma patients) and tumor tissues were assessed. Upregulation of FSHR, ALKBH5, Snail was observed in ovarian tumor. IHC analysis also showed that decreased expression of E-cadherin, but increased expression of N-cadherin in ovarian tumor (Figure 6C and Figure S5D, E). TCGA data reveals the positive correlation between FSH, ALKBH5 and Snail expression in ovarian cancer (Figure 6D, E). Survival analysis showed both ALKBH5 and Snail higher expression was related to shorter overall survival times in ovarian cancer patients (Figure S5F, G). In human specimen, we also confirmed that FSHR, ALKBH5 and Snail protein expression showed significant upregulation in ovarian tumors, compared to normal ovarian tissue (Figure 6F), m6A content in total mRNA was higher than those in ovarian tumors (Figure 6G). Interestingly, we also found that METTL14 and YTHDF2 expression were decreased in ovarian tumor (Figure S5H). These results identified the role and strong correlation between FSHR, ALKBH5 and Snail in ovarian cancer progression.

## Discussion

Although the "gonadotropin theory" of ovarian cancer pathogenesis remains a subject of controversy 14, 44, recent immunotherapies targeting FSHR have demonstrated high level of safety and efficacy in ovarian cancer treatment 11, 12. Therefore, confirming the role of FSH in ovarian tumors and its underlying mechanisms would be essential for further optimizing the clinical application of FSHR immunotherapies. Our study revealed that FSH promoted metastasis in ovarian tumors through the EMT process, with ALKBH5-induced demethylation of m6A modifications in *Snail* mRNA playing a critical role in this procedure. This suggests that regulation on the reversible m6A modifications could be an effective approach to explore FSH-induced ovarian tumor metastasis.

Apart from its role in maintaining reproductive system development and regulating the function of extragonadal organs, FSH is associated with various tumor processes, including breast cancer 45, non-functional pituitary adenomas 46, and ovarian cancer 47. With the discovery of FSHR in normal ovarian epithelial cells and ovarian cancer cells 4, the role of FSH in the ovarian tumor process induced by abnormal epithelial cell development has progressively attracted research interest. Our study found that, in tumor tissues from 160 patients with different stage of ovarian cancer, the expression of FSHR was found to be correlated with metastasis. Using the epithelial ovarian cancer cell line SK-OV3 and a nude mouse ovarian intraperitoneal metastasis model, we confirmed that FSH induced EMT and promoted ovarian tumor metastasis. Other than cell migration and invasion, FSH participated in ovarian tumor processes by regulating cell proliferation, apoptosis, and promoting tumor angiogenesis 48. Our study, along with others, suggested that monitoring and intervening in hormone levels could constitute a safe and effective approach for prevention and treatment of hormone-related cancers.

Tumor metastasis is a major cause of death in ovarian cancer patients 16. EMT is the initial step where cancer epithelial cells acquire the ability to move, leading to tumor metastasis 49. Recent studies suggested that small molecule targeted therapy of EMT in tumor epithelial cells indicate a potential treatment for tumor metastasis 50. In our study, FSH was shown to inhibit E-cadherin and increase N-cadherin, contributing to EMT and the metastasis of epithelial ovarian cancer cells *in vivo* and *in vitro*. Consistently, in tissues of nonfunctioning pituitary tumors, the concentrations of FSH were inversely associated with E-cadherin expression 51. Moreover, research has reported that FSHR is present in metastases to lymph nodes, brain, bones, and liver, which were originated from breast, lung, prostate, and renal cancers 52. Together, these results suggested that FSH influences tumor metastasis, and small molecule targeted therapy of FSH/FSHR holds promise as a safe and effective approach for treating cancer metastasis.

In tumor microenvironment, cancer and immune cells release cytokines, growth factors, and chemokines towards surrounding epithelial cells. This action activates EMT transcription factors (Snail, Slug, Twist, ZEB1, and ZEB2) and initiates EMT in the epithelial cells 53. Snail is a classical transcription factor of EMT, and its activation is correlated with tumor pathological grading, lymph node metastasis, and poor prognoses of tumor metastasis 54. Research has demonstrated that transcriptional activity of the *Snail* promoter is directly modulated by various growth factors and signaling molecules 55. However, our results indicated that FSH has no impact on the transcriptional activation of *Snail* promoter. Furthermore, post-translational modifications, including ubiquitination 56, phosphorylation 57, and m6A modification 30, regulate the mRNA stability and protein expression of Snail, thereby impacting EMT. Our study confirmed that FSH increased Snail protein expression by stabilizing *Snail* mRNA via m6A modification. This result may explain the findings of Yang *et al* who observed FSH upregulating Snail protein in ovarian cancer 58. Additionally, our discovery is corroborated by the observation that m6A modification in *Snail* mRNA was regulated by the methyltransferase METTL3 30 in nasopharyngeal carcinoma and the demethylase FTO 59 in ovarian cancer. Importantly, our research identified the CDS region of *Snail* mRNA was the site of FSH-induced m6A modification. These results suggested that by leveraging the reversible nature of m6A regulation, regulation on m6A modification at specific sites on Snail could be a potential approach to treat tumor metastasis.

In tumor metastasis, m6A modification is linked to the tumor immune microenvironment 60, glucose metabolism of cancer cells 61, and migration and invasion capabilities 62. Yu *et al* discovered m6A modifications in the EMT transcription factor Slug in head and neck squamous carcinoma 29. Similarly, Lin *et al* identified m6A modifications in the EMT transcription factor ZEB1 in triple-negative breast cancer 31. These observations aligned with our conclusion that the m6A modification in the EMT transcription factor Snail plays a pivotal role in FSH-induced EMT, contributing to ovarian tumor metastasis. Furthermore, we confirmed that the upregulation of demethylase ALKBH5 is a key factor leading to FSH-induced EMT, primarily by increasing Snail protein expression. However, studies on renal fibrosis suggested that the drug Genistein inhibited the occurrence of EMT by downregulating Snail through ALKBH5 63. These varied regulatory outcomes suggested that the influence of ALKBH5 on Snail may be contingent on distinct tissues or cell types, exhibiting diverse roles in various diseases 64.

ALKBH5 is implicated in the proliferation, migration, invasion of cancer cell, and tumor metastasis 65. Our research for the first time demonstrated that ALKBH5 promotes EMT and facilitates intraperitoneal metastasis in ovarian cancer cells. This finding aligned with the discovery by Hu *et al* in clear cell renal cell carcinoma, showcasing the role of ALKBH5 in tumor metastasis through EMT 66, and consistent with the observation by Sun *et al* reporting the association of ALKBH5 with ovarian tumor-related lymphatics and lymphatic metastasis 23. Moreover, we clarified the underlying mechanism by which ALKBH5 promotes EMT by increasing *Snail* mRNA stability through m6A modification. The stability of *Snail* mRNA is crucial in the context of tumor metastasis, as previously confirmed in lung cancer 67. Therefore, intervening in *Snail* mRNA stability through pathways like ALKBH5 may represent a potential approach to decelerate tumors metastasis. Simultaneously, several studies indicate that ALKBH5, through regulating the stability of downstream gene mRNA, is involved in the progress of non-tumor diseases, particularly infertility resulting from abnormal reproductive system development 40. These data indicated that ALKBH5 stabilizes the target mRNA, directly regulating tumor cells or influencing the tumor microenvironment, thereby playing an essential role in tumor metastasis.

Studies have indicated that the transcriptional process of ALKBH5 is jointly regulated by epigenetic factors and transcription factors 64. Our experiments confirmed that the transcription factor CREB, as a classic downstream molecule of FSH, upregulates the level of ALKBH5 in ovarian tumors, ultimately leading to EMT occurrence. This finding was analogous to the results obtained by Guo *et al* in colorectal cancer cells, where activating the CREB signaling pathway significantly increased EMT in colorectal cancer cells 68. Research has shown that FSH increased promoter activity of CREB in ovarian epithelial cells 69. Moreover, CREB highly expressed in various tumors, including ovarian cancer 70, suggesting that CREB, as a responsive hormone activity intermediate regulator, plays a crucial role in tumors. The studies mentioned above suggested that targeting the CREB and downstream m6A-related signaling pathways in hormone-related tumor processes may represent a potential strategy for retarding tumor progression.

While this study provided a new perspective on FSH regulation of ovarian tumor metastasis via m6A modification, limitation in this study should be acknowledged. Although we have observed that FSH decreased METTL14 expression, the 'reader' of m6A, the role of METTL14 in EMT of ovarian cancer remains to be investigated. The role of Snail-regulated m6A modification in FSH-induced tumor metastasis has not been validated in animal experiments. Although m6A inhibitors for *in vivo* study, *e.g.,* 3-deazaadenosine, are commercially available, the specificity of this inhibitor for m6A modification in *Snail* mRNA remained limited. Theoretically, development of mice with mutated methylation sites in *Snail* could address this limitation, however, that will be future work for our next project.

In conclusion, this study demonstrated that FSH activates CREB, leading to increased expression of ALKBH5. Subsequently, ALKBH5 induces m6A demethylation in the CDS region of *Snail* mRNA, enhancing mRNA stability and upregulating protein expression of Snail. This cascade of events induced EMT and ovarian cancer metastasis. Our findings suggested that disrupting m6A modification through intervention in FSH and its downstream signaling pathways could provide a novel therapeutic approach for ovarian tumor metastasis.

## Conclusions

Overall, this study indicated that FSH increased *ALKBH5* mRNA and protein expression via CREB activation. ALKBH5 triggers *Snail* mRNA m6A demethylation on its CDS region to increase Snail protein expression, thus induces EMT and ovarian cancer metastasis. It is anticipated that blocking FSH/FSHR and downstream signaling pathway via m6A alteration would provide a novel therapeutic avenue for ovarian tumor metastasis.

## Materials and methods

### Cell culture studies

Human epithelial ovarian cancer cell lines (SK-OV3, ES-2, A2780) and HEK293T cell line were purchased from the American Type Culture Collection (VA, USA). SK-OV3 and ES-2 cells were cultured in McCoy's 5A medium (#X054B1, BasalMedia) supplemented with 10% (v/v) heat-inactivated fetal bovine serum (FBS, #10270-106, Gibco), 100 unit/mL penicillin and 100 µg/mL streptomycin (P/S, #SV30010, Hyclone), while A2780 and HEK293T cells were grown in DMEM (#21041025, Gibco) supplemented with 10% (v/v) FBS and P/S. Opti-MEM (#31985) were acquired from Gibco (Waltham MA USA). Cells were cultured in a humidified environment of 95% air, 5% CO_2_ at 37°C. Experiments were performed when the cells reached 90% confluence.

### Clinical specimens

5 pairs of epithelial ovarian cancer and normal ovarian tissue were obtained from the Guangzhou First People's Hospital, normal ovarian tissues samples were obtained from donors during surgery for other benign gynecological diseases. Diagnosis of normal and tumor tissues was performed by experienced pathologists. All patients were informed prior to the samples donation and had provided their written informed consent. The research in this work followed the principles of Helsinki Declaration and was approved by the Institutional Review Board of the Guangzhou First People's Hospital.

### Tissue microarray

The tissue microarray (TMA) of ovarian tissues was obtained from Shanghai WellBio Technology. The TMA in Table 1 contained 160 ovarian tumor samples, TMA in Table 2 contained 88 ovarian tumor samples and 8 normal ovarian tissues samples, the patients in two TMA were recruited in Tongxu First Hospital, Kaifeng, Henan, China from 2015 to 2021. The TMA was sectioned at 5 µm thickness. Adjacent sections were used to assess the expression of ALKBH5 and Snail in the TMA using immunohistochemistry as described above. FSHR, ALKBH5 and Snail expression were independently evaluated by two experienced pathologists using double-blind approach. Tissue sections were divided into four groups: negative, weak, medium and strong grades. Negative and weak staining were classified as low expression, according to the protein expression, while medium and strong staining were classified as high expression.

### Animals

Female Balb/c nude mice were obtained from Beijing Vital River Laboratory Animal Technology (Beijing, China). All mice were housed in a temperature-controlled room (Physical Containment Level 2, PC2) on a 12-hour light and dark cycle at Guangzhou Medical University Animal Center and were allowed access to water and food ad libitum. All animal experiments were carried out according to the Guide for the Care and Use of Laboratory Animals outlined by the National Institutes of Health (NIH) and conformed to the guidelines of Animal Ethics Committees of Guangzhou Medical University (Protocol: 2020-077).

Female Balb/c nude mice at 8 weeks of age were anesthetized using pentobarbital sodium (1%, 80 mg/kg), followed by bilateral ovariectomy (OVX), where two sides of ovaries were surgically excised with a single surgical incision performed by one surgeon as previously described 71. After surgery, mice were allowed two weeks of recovery period before subjected to further experiments.

To established tumor xenotransplants, recovered OVX Balb/c nude mice were randomly divided into two groups. SK-OV3 cells stably expressing control shRNA (*n* = 8) or ALKBH5 shRNA (*n* = 8) were harvested and counted, respectively. SK-OV3 (2×10^6^ cells per mouse) were resuspended in 100 μL PBS was then intraperitoneal injected into each mouse according to its respective group. One week after, PBS (vehicle control, *n* = 4 in each group) or recombinant FSH (3 IU per mouse, #F4021, Merck) dissolved in PBS (FSH, *n* = 4 in each group) was subcutaneously injected into mice every day for 14 weeks, according to previous study 71. Throughout the study, animal body weight was monitored in a weekly base.

IVIS Lumina XRMS was used to track the progression of *in vivo* tumor peritoneum metastasis weekly one week after the intraperitoneal injection of SK-OV3 cells. OVX tumor bearing mice were anesthetized using isoflurane (4% for induction, 2% for maintenance). D-luciferin (15 mg/mL, 150 mg/kg, #122799, PerkinElmer) was intraperitoneally administrated into mice 10 minutes before IVIS imaging of luminescence (5 minutes exposure). The data were analyzed using Living Image software.

### Cell transfection and lentiviral infection

The Snail siRNA was purchased from Santa Cruz Biotechnology (Santa Cruz, CA), and ALKBH5 siRNA (CCATGACGTCCCGGGACAACTATAA) was constructed and obtained from Invitrogen. siRNAs (40 nM diluted in 1 mL Opti-MEM) were transfected to SK-OV3 cells using Lipofectamine RNAiMAX (#13778150, Invitrogen). The plasmid of wild-type (pENTER-ALKBH5) and mutated ALKBH5-H204A (pENTER-ALKBH5-H204A) was obtained from WZ Biosciences Inc, while plasmid of wild-type (pSLenti-Snail) and mutated Snail (pSLenti-Snail-mut, adenosine bases in m6A consensus sites replaced by cytosine nucleotides) was constructed and obtained from OBiO Technology. Plasmid (2 µg diluted in 1 mL Opti-MEM) was transfected to SK-OV3 cells using Lipofectamine 3000 Transfection Reagent (#L3000015, Invitrogen). The shRNA of human ALKBH5 was cloned into a lentiviral vector (pLent-3in1-shRNA-CMV-copGFP-P2A-Puro) for knockdown. Sequences of the shRNAs were as followed, stably transfected SK-OV3 cells were selected using puromycin (#ST551, Beyotime, China): siRNA1: GTCCTTCTTTAGCGACTCT; siRNA2: CCATGACGTCCCGGGACAACTATAA; siRNA3: GCTCAGTGGATATGCTGCTGATGAA.

### Immunofluorescence assay

SK-OV3 cells were cultured on glass coverslips (#801009, Nest) in six-well plates, fixed with 4% paraformaldehyde (#430012, Asegene), and permeabilized with 0.3% Triton X-100 (#DH351-1, Ding Guo Prosperous). The cells were then blocked in 5% BSA and incubated with primary antibodies against E-cadherin (1:1000, #60335-1-Ig, Proteintech), N-cadherin (1:1000, #22018-1-AP, Proteintech), ALKBH5 (1:1000, #16837-1-AP, Proteintech) and CREB (1:1000, #9104S, Cell Signaling Technology) at 4°C overnight, followed by FITC-conjugated secondary antibodies incubations (1:1000; Alexa Fluor® 555 Conjugate, #4409S, Cell Signaling Technology; 1:1000, Alexa Fluor™ Plus 488, #A32731, Invitrogen) at room temperature for 1 hour. Nuclei were counterstained with DAPI (1:5000, #4083S, Cell Signaling Technology). Finally, cells on glass coverslips were mounted on glass slides (#188105W, Citoglas) with antifade mounting reagent (#S2100, Asegene). The fluorescent signal was visualized using Zeiss microscope (LM980, Zeiss).

### Western blotting and antibodies

After treatment, tissue or cells were washed with ice-cold PBS, lysed with lysis buffer (100 mM Tris-HCl (pH=6.8), 4% SDS, 20% glycerol, 1× protease inhibitors cocktail (Sigma, #P8340), 1× phosphatase inhibitors cocktail (Sigma, #P0044), 1 mM sodium orthovanadate, 1 mM NaF, and 1 mM phenylmethylsulfonylfluoride, PMSF). Lysate were scraped, harvested, denatured with boiling water, then centrifuged. The resulting supernatant were collected, and protein contents were determined using BCA assay (Thermo scientific, #23235). 25 µg protein was then added onto a 10% SDS-PAGE gel for separation by electrophoresis. Separated protein was then transferred to PVDF membrane, blocked with 5% BSA at room temperature for 60 min, and incubated with corresponding primary antibodies at 4°C overnight. Membrane was then washed with TBST and incubated with corresponding HRP-conjugated secondary antibodies at room temperature for 1 hour. Membrane was washed, while protein was detected and visualized using Enhanced Chemiluminescence reagent (#WBKLS0500, Millipore) and Imaging Systems (Amersham Imager 600, GE).

All antibodies used in this study were as follows: anti-FSHR (#22665-1-AP), anti-E-cadherin (#20874-1-AP), anti-N-cadherin (#22018-1-AP), anti-Snail (#13099-1-AP), anti-ZEB1 (#66279-1-Ig), anti-ZEB2 (#14026-1-AP), anti-ALKBH5 (#16837-1-AP), anti-Vimentin (#10366-1-AP), anti-GAPDH (#60004-1-Ig) were obtained from Proteintech. Anti-Slug (#9585S), anti-FTO (#45980S), anti-p-CREB (#9198S), anti-CREB (#9104S), anti-Twist (#90445), anti-Mouse IgG HRP-linked (#7076S), anti-Rabbit IgG HRP-linked (#7074S) were obtained from Cell Signaling Technology. Anti-β-actin (#sc-81178) was obtained from Santa Cruz. Anti-FTO (#ab124892), anti-METTL3 (#ab195352), anti-YTHDF1 (#ab99080), anti-YTHDF3 (#ab103328) were obtained from Abcam. Anti-YTHDF2 (#ABE542) was obtained from Millipore. Anti-METTL14 (#HPA038002) was obtained from Sigma. Before using, all primary antibodies were diluted in 1:2000 with 5% BSA, and the corresponding HRP-conjugated secondary antibodies were diluted in 1:10000 with 5% BSA.

### RNA isolation and RT-qPCR

RNA extraction was performed using SteadyPure Universal RNA Extraction Kit (#AG21017, Accurate Biology) according to the manufacturer's instruction. Complementary DNA was prepared by reverse transcription using Evo M-MLV reverse transcriptase (#AG11706, Accurate Biology) following the user's manual. Real-time quantitative PCR was performed on thermal cycler (CFX96, Bio-Rad) using the SYBR Green probe (#AG11701, Accurate Biology). RNA transcripts were determined by relative to GAPDH 72 using the 2^-△△^Ct method as described in previous publication 73. The detailed information of PCR primers was listed in the **Supplementary Table 1**.

### Wound-healing assay

SK-OV3 cells were seeded onto a six-well plate with culture-insert (ibidi, #80496), which provide a defined cell-free gap for wound healing assays. 24 hours after seeding, culture-insert was removed. Cells were gently washed twice with PBS, cultured with FBS-free medium and ready for the subsequent treatments. 0 and 48 hours after treatment, three random fields of the wound healing area were imaged and used to determine cell migration.

### Transwell invasion assay

SK-OV3 cell suspension (2×10^4^ cells in FBS-free culture media) were added into the upper chamber of Matrigel-coated inserts (#356234, BD Bioscience). The Matrigel-coated inserts was then carefully transferred to the 24-well plate containing 10% FBS culture media for cell invasion. After pre-determined invasion period of 48 hours, inserts were carefully washed with PBS without disturbing the Matrigel layer, then fixed with 4% paraformaldehyde and stained with 0.1% crystal violet (#G1064, Solarbio). Non-invaded cells on the upper chamber were carefully removed. Insert were then placed on a slide for imaging. Five random fields were captured and used for cell counting.

### Promoter activity and luciferase reporter assay

DNA fragment of ALKBH5 promoter (2000 bp) was cloned into pGL3 promoterless vector upstream of the luciferase gene to construct a recombinant plasmid of pGL3-Basic-ALKBH5-luc. The plasmids (800 ng diluted in 0.5 mL Opti-MEM) of pGL3-Basic-ALKBH5-luc and pRL-TK (80 ng diluted in 0.5 mL Opti-MEM) were then co-transfected into HEK293T cells using Lipofectamine 3000 Transfection Reagent (Invitrogen). After drug treatment for the transfected cells, promoter activity was assessed by measuring the luciferase activity using the Promega Reporter Assays (#E1910, Promega) according to the manufacturer's instruction (SPARK 10M, TECAN). Promoter activity of pGL3-Basic-ALKBH5-luc was then determined by relative to pRL-TK promoter activity using the comparative method.

### Immunohistochemistry

Human ovarian tumor, health ovarian tissues, and SK-OV3 xenografts were collected, formalin-fixed and paraffin-embedded. Tissue paraffin blocks were then sectioned at 4 µm thickness (RM2016, Leica biosystems) and placed on a glass slide. Tissue sections were deparaffined using in xylene and rehydrated in serial dilution of ethanol from 100% to 70%. Rehydrated tissue sections were incubated with 0.3% H_2_O_2_ to quench the endogenous peroxidase, and then blocked with 3% BSA (#G5001, Servicebio). Primary antibodies of FSHR (1:200, #22665-1-AP, Proteintech), ALKBH5 (1:200, #ab195377, Abcam), Snail (1:200, #SC-271977, Santa Cruz), anti-E-cadherin (1:200, #20874-1-AP, Proteintech), anti-N-cadherin (1:200, #22018-1-AP, Proteintech), Ki-67 (1:200, #12202S, Cell Signaling Technology) and isotype antibodies (1:200, #31235, Cell Signaling Technology) were used to treat the tissue section at 4 °C overnight, followed by the correspond HRP-conjugated secondary antibodies. After washing, protein signal was visualized using DAB (#GK500710, Gene Tech). Stained sections were imaged using a bright field microscope (Leica CS2, Leica biosystems). Image J software was used to quantify the average optical density (AOD).

### Analysis of m6A content

Total RNA was extract using SteadyPure Universal RNA Extraction Kit (#AG21017, Accurate Biology). Total m6A content was measured using the m6A RNA Methylation Assay Kit (#ab185912, Abcam). Briefly, 80 ul of binding solution and 200 ng of sample RNA were add into pre-coated plate and incubate at 37°C for 90 min for RNA binding. The dissociated RNA was discarded using washing buffer. After incubation with capture antibody, detection antibody and enhancer solution in sequence, m6A modification RNA was captured. Finally, the wells were incubated with developer solution in the dark, then stop solution was used to stop the reaction, m6A content was quantified using a microplate reader at 450 nm wavelength.

### Analysis of *Snail* mRNA methylation level

Total RNA was extracted from cells using Trizol (#15596081, Ambion) according to the manufacturer's instruction. mRNA was separated from total RNA according to the protocol of the GenElute™ mRNA Low Dose Preparation Kit (MRN70, Sigma). Then the above mRNA was concentrated with glycogen (AM9510, Invitrogen), and the mRNA fragment was processed. RNA was incubated with m6A antibody for immunoprecipitation using Magna methylated RNA immunoprecipitation m6A Kit (MAGNAPIP, Millipore), qRT-PCR was performed using 2 µL m6A IP RNA as described above. The detailed information of the PCR primers is listed in the **Supplementary Table 1**.

### Statistical analysis

The individual data was expressed as mean ± standard error of the mean (SEM) from at least three independent experiments unless otherwise specified. Unpaired Student's t-test and Mann-Whitney tests were used in the comparison of two groups, while one-way ANOVA was used for multiple comparison. The Pearson's χ2, Pearson's corrected χ2, and Fisher's exact tests were used to analyze the association between ALKBH5/Snail expression, FSHR expression and clinicopathological characteristics. Statistical analyses were performed with SPSS 17.0. A *P*-value of < 0.05 was considered to be statistically significant. *P < 0.05, **P < 0.01, ***P < 0.001, ns. not significant.

## Supplementary Material

Supplementary figures and table.

## Figures and Tables

**Table 1:** Association between FSHR and clinicopathologic characterizations in ovarian cancer

	FSHR expression			
Characteristics	Patients	Low	High	*P*-value
Ages				
≤	57	24	33	0.49^a^
>50	103	45	58	
T Stage				
T1+T2	119	51	68	0.525^a^
T3+T4	41	18	23	
Node Metastasis				
Absent	123	58	65	0.045^a^
Present	37	11	26	
CA-125				
≤	47	17	30	0.166^a^
>200	113	52	61	
Tumor Grade				
Ⅰ	24	14	10	0.061^a^
Ⅱ	33	18	15	
Ⅲ	103	38	65	
Histological Type				
Serous	51	24	27	0.654^b^
Mucinous	107	45	62	
Others	2	1	1	

Number in the table represented the case number of patients. a: Pearson Chi-Square test; b: Fisher's exact test;

**Table 2:** Association between ALKBH5, Snail, and clinicopathologic characterizations in ovarian cancer

	ALKBH5 expression				Snail expression				
Characteristics	Patients	Low	High	*P*-value	Patients	Low	High		*P*-value
Ages									
≤	24	18	6	0.218^a^	24	16		8	0.019^a^
>50	72	44	28		72	43		29	
T Stage									
T1+T2	50	36	14	0.019^a^	50	30		20	< 0.001^a^
T3+T4	38	18	20		38	21		17	
Node Metastasis									
Absent	7	7	0	0.04^b^	7	6		1	0.058^c^
Present	81	47	34		81	45		36	
Distant Metastasis									
Absent	55	36	19	< 0.001^a^	55	34		21	< 0.001^a^
Present	33	18	15		33	17		16	
Peritoneal Seeding									
Absent	51	39	12	0.001^a^	51	33		18	< 0.001^a^
Present	37	15	22		37	18		19	
Ascites									
Absent	81	52	29	< 0.001^c^	81	48		33	< 0.001^c^
Present	7	2	5		7	3		4	
Ovarian Tissue									
Normal	8	8	0	0.047^d^	8	8		0	0.022^d^
Cancer	88	54	34		88	51		37	
Primary Tumor Origin									
Ovarian	81	47	34	0.04^b^	81	45		36	0.038^b^
Others	7	7	0		7	6		1	
Histological Type									
Serous	49	24	25	0.037^d^	49	24		25	0.198^d^
Mucinous	9	8	1		9	8		1	
Endometrioid	14	6	8		14	6		8	
Clear cell carcinoma	9	9	0		9	7		2	
									

Number in the table represented the case number of patients. a: Pearson Chi-Square test; b: Fisher's exact test; c: Continuity correction test; d: Mann-Whitney Test;

## Abbreviations

FSH: follicle-stimulating hormone
EMT: epithelial-mesenchymal transition
m6A: N6-methyladenosine
ALKBH5: AlkB Homolog 5, RNA Demethylase
qRT-PCR: quantitative real time PCR
m6A-RT-PCR: methylated RIP-qPCR
FSHR: follicle stimulating hormone receptor
ActD: actinomycin D
CHX: cycloheximide
OVX: ovariectomized
CCK8: cell counting kit-8
TMA: tissue microarray
IHC: immunohistochemistry
CREB: cAMP response element-binding protein
