# Supplementary material for: FSH induces EMT in ovarian cancer via ALKBH5-regulated Snail m6A demethylation
Source: Theranostics. 2024 Mar 3;14(5):2151–66. doi: 10.7150/thno.94161 (PMC10945345; doi:10.7150/thno.94161)
Supplement: Supplementary file 1 — Supplementary figures and table. [file thnov14p2151s1.pdf]

Supplementary table 1. Detailed information PCR primers

Primer name	Genbank Accession No.	Forward	Reverse	Product size (bp)
Snail	NM_005985.4	GGCAATTTAACAATGTCTGAAAAGG	GAATAGTTCTGGGAGACACATCG	105
ALKBH5	NM_017758.4	AGTTCCAGTTCAAGCCTATTCG	TGAGCACAGTCACGCTTCC	78
Snail-m6A	NM_005985.4	AGGTCAGCTCTGCCACCCT	TCCCACCGTCTCGACTGGA	108
E-cadherin	NM_004360.5	CGAGAGCTACACGTTCACGG	GGGTGTCGAGGGAAAAATAGG	119
GAPDH	NM_002046.7	GGAGCGAGATCCCTCCAAAAT	GGCTGTTGTCATACTTCTCATGG	197

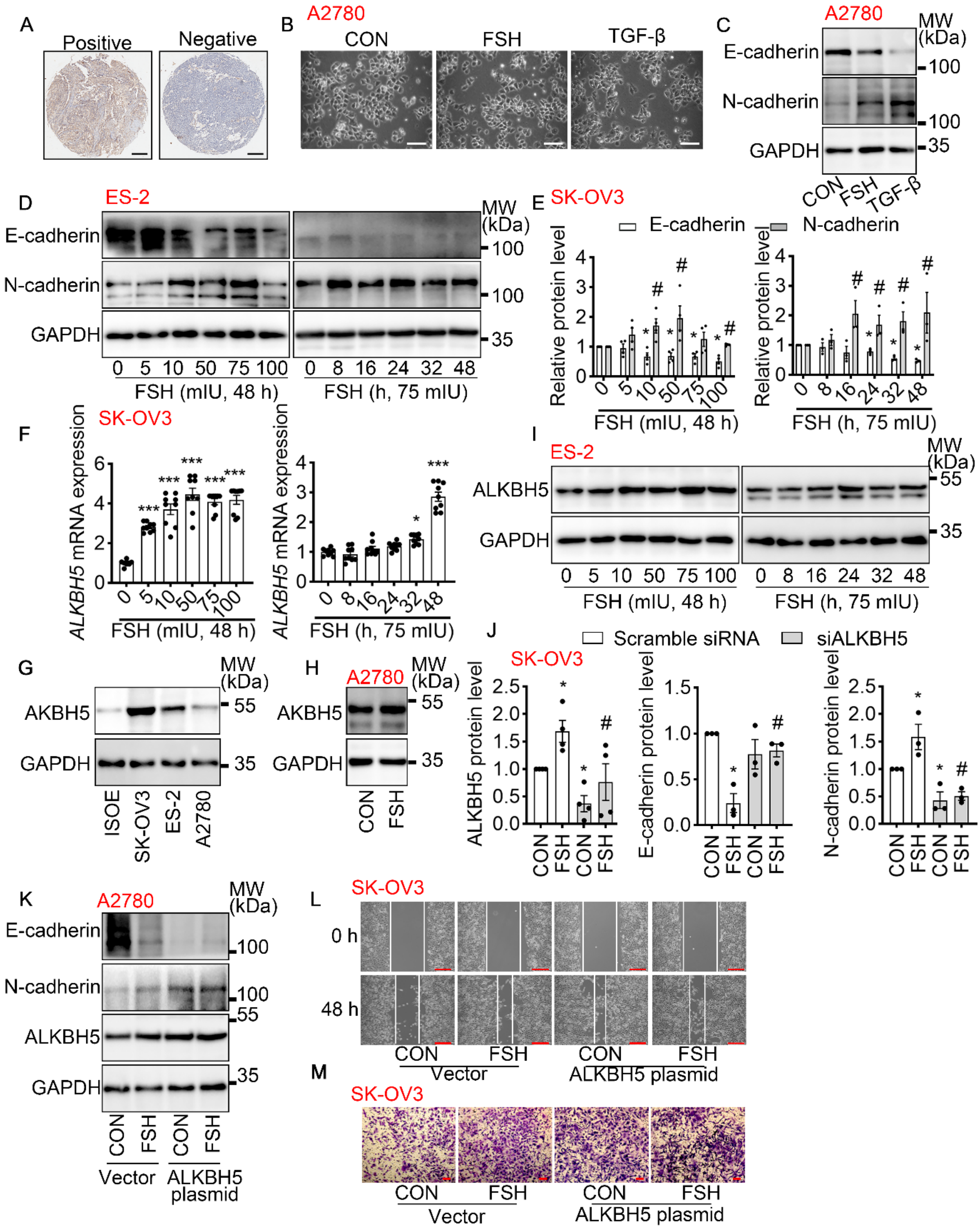

Supplementary figure 1. FSH induced EMT in epithelial ovarian cancer cells via ALKBH5. **(A)** IHC of FSHR on tissue microarray of ovarian tumor. Scale bar = 250 μm. **(B)** Cellular morphology of A2780 cells treated with FSH (75 mIU) and TGF-β (10 ng/μl) for 48 hours. Scale bar = 25 μm. **(C)** Western blots of E-cadherin and N-cadherin in A2780 cells treated with FSH (75 mIU) and TGF-β (10 ng/μl) for 48 hours. **(D)** Western blots of E-cadherin and N-cadherin in ES-2 cells treated with different concentrations of FSH for various time points. **(E)** Quantification of Western blot for E-cadherin and N-cadherin expression in SK-OV3 cells treated with different concentrations of FSH for various time points (n = 3 - 4), data was analyzed

by Mann-Whitney U test. *P < 0.05 *versus* CON (E-cadherin), #P < 0.05 *versus* CON (N-cadherin). **(F)** qRT-PCR of *ALKBH5* mRNA in SK-OV3 cells treated with different concentrations of FSH for various time points (n = 9), data was analyzed by one-way ANOVA test. *P < 0.05, ***P < 0.001 *versus* CON. **(G)** Western blots of ALKBH5 expression in ISOE-80 (ISOE), SK-OV3, ES-2, A2780 cell lines. **(H)** Western blots of ALKBH5 expression in A2780 cells with FSH (75 mIU) treatment for 48 hours. **(I)** Western blots of ALKBH5 expression in ES-2 cells were treated with different concentrations of FSH for various time points. **(J)** Western blot quantification of ALKBH5, E-cadherin and N-cadherin in SK-OV3 cells treated with FSH after transfection of scramble or ALKBH5 siRNA (n = 3 - 4), data was analyzed by Mann-Whitney U test. *P < 0.05 *versus* CON group transfected with scramble siRNA, #P < 0.05 *versus* FSH group transfected with scramble siRNA. **(K)** Western blots of ALKBH5, E-cadherin and N-cadherin in A2780 cell treated with FSH (75 mIU, 48 h) after transfection of vector or ALKBH5 plasmid. **(L)** Representative images of wound healing in SK-OV3 treated with FSH (75 mIU, 48 h) after transfection of vector or ALKBH5 plasmid. Scale bar = 100 μm. **(M)** Representative images of Transwell invasion in SK-OV3 treated with FSH (75 mIU, 48 h) after transfection of vector or ALKBH5 plasmid. Scale bar = 250 μm.

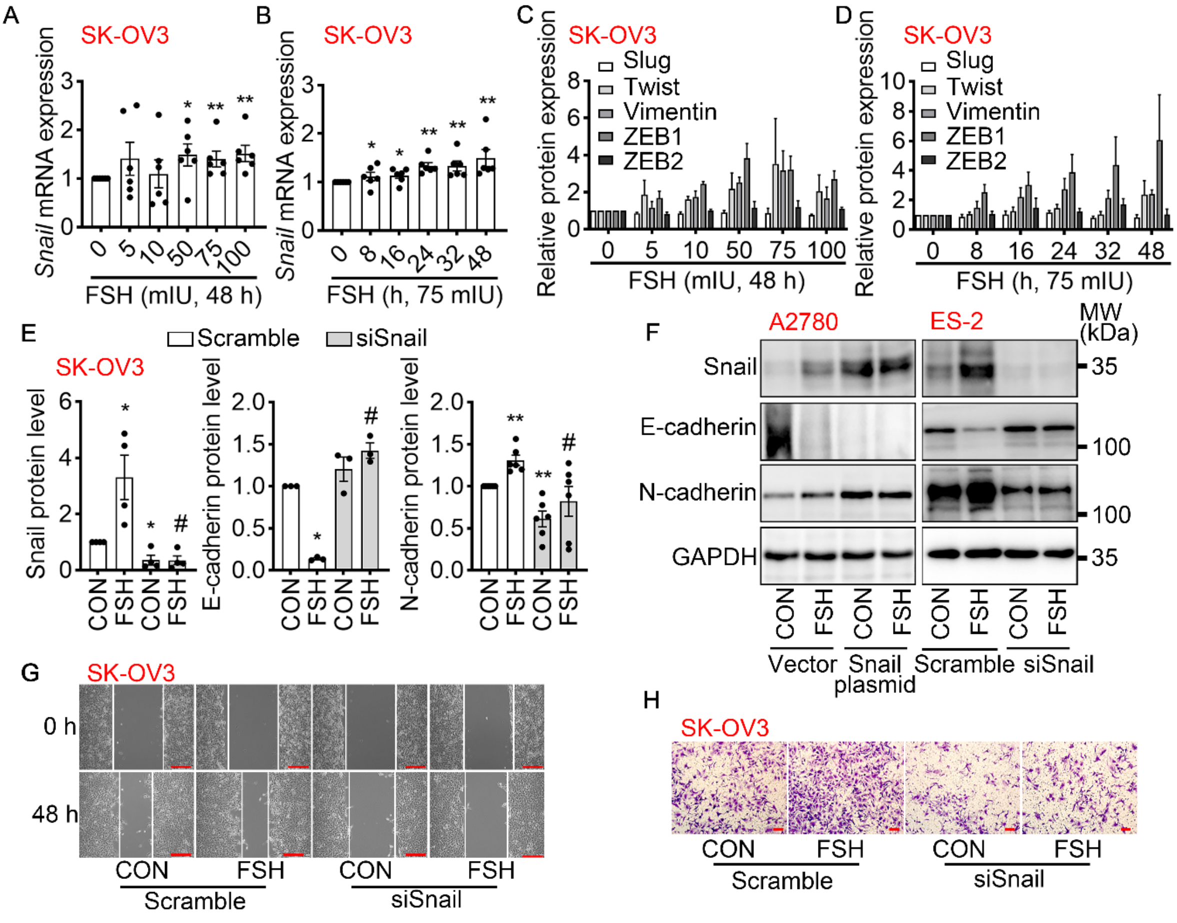

Supplementary figure 2. Snail is a critical EMT transcription factor in FSH induces EMT progression. **(A-D)** SK-OV3 cells treated with different concentrations of FSH for various time points. **(A, B)** qRT-PCR of *Snail* mRNA transcripts, $n = 6$ per group, data was analyzed for statistical difference by Mann-Whitney U test. $^{*}P < 0.05$, $^{**}P < 0.01$. (C, D) Quantification of Western blot for Slug, Twist, Vimentin and ZEB1/2 ($n = 3$). **(E)** Quantification of Western blot for Snail, E-cadherin and N-cadherin in SK-OV3 cells treated with FSH (75 mIU, 48 h) after transfection of scramble or Snail siRNA (siSnail). ($n = 3 - 5$), data was analyzed by Mann-Whitney U test. $^{*}P < 0.05$, $^{**}P < 0.01$ *versus* CON group transfected with scramble siRNA, $\#P < 0.05$ *versus* FSH group transfected with scramble siRNA. **(F)** Western blots of Snail, E-cadherin and N-cadherin in A2780 (left) or ES-2 cells (right) treated with FSH (75 mIU, 48 h) after transfection of Snail plasmid or Snail siRNA. **(G, H)** SK-OV3 cells were treated with FSH (75 mIU, 48 h) after transfection of scramble or Snail siRNA. **(G)** Representative images of wound healing (scale bar = 100 μm) and **(H)** Transwell invasion (scale bar = 250 μm).

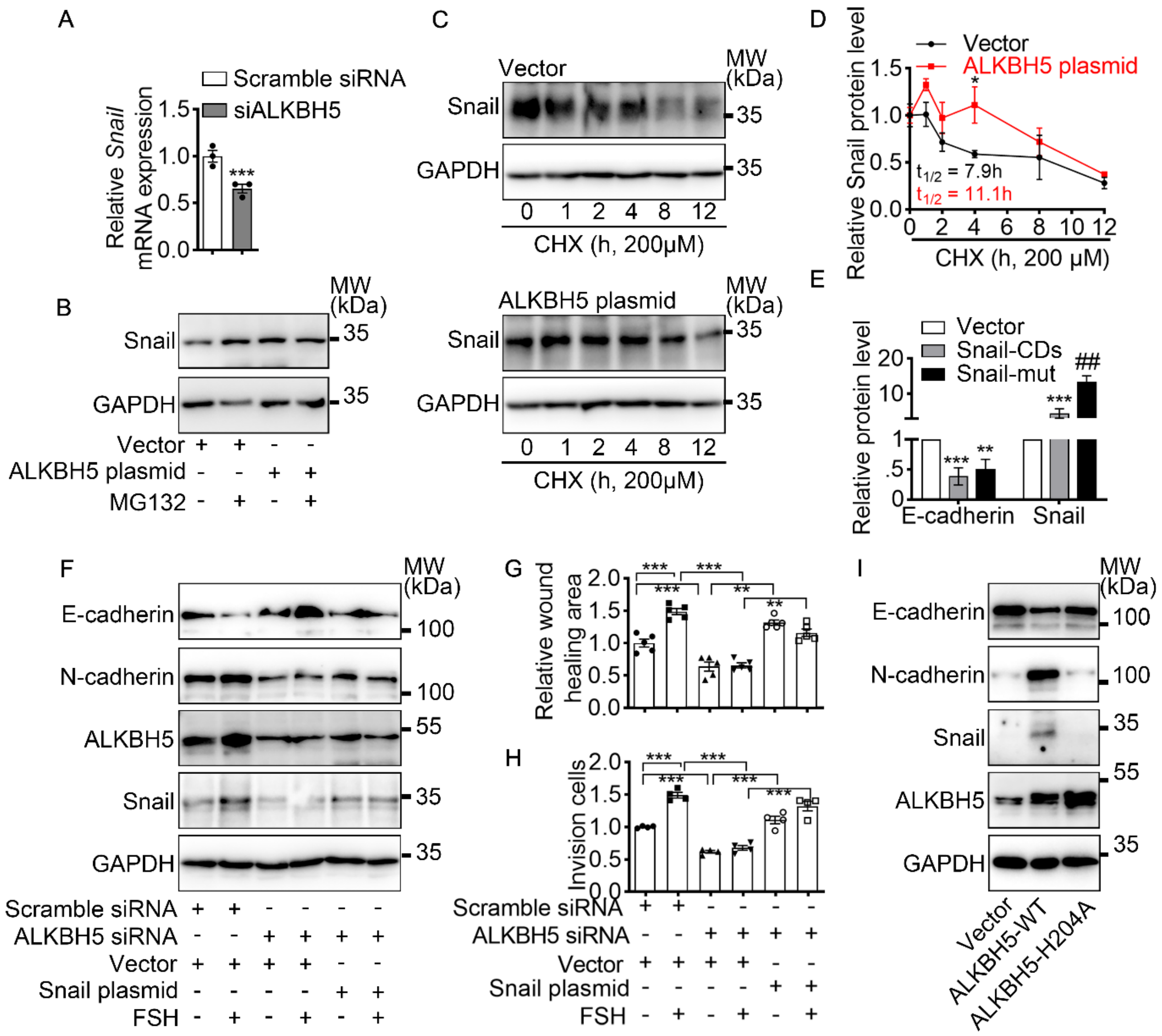

Supplementary figure 3. Increased Snail expression induced by ALKBH5 is essential for FSH-triggered EMT. **(A)** qRT-PCR of *Snail* mRNA transcripts in SK-OV3 cells transfected with Scramble and ALKBH5 siRNA (siALKBH5), (n = 3). Data was analyzed by two-tailed unpaired t test. ***P < 0.001. **(B)** Western blots of Snail in SK-OV3 cells transfected with vector or ALKBH5 plasmid with treatment of proteasome inhibitors MG132. **(C)** Western blots of Snail in SK-OV3 cells transfected with Vector or ALKBH5 plasmid with treatment of cycloheximide (CHX), quantitative analysis was present in the right panel **(D)**. **(E)** Quantification of Western blot for E-cadherin and N-cadherin in SK-OV3 cells transfected with Vector, Snail-CDs and Snail-mut plasmid (n = 3), data was analyzed by one-way ANOVA test. **P < 0.01, ***P < 0.001 *versus* Vector, ##P < 0.01 *versus* Snail-CDs. **(F-H)** SK-OV3 cells were solely transfected with ALKBH5 siRNA or co-transfected with ALKBH5 siRNA and Snail plasmid followed by treatment of FSH. **(F)** Protein expression of E-cadherin, N-cadherin, ALKBH5 and Snail were assessed by Western blot. **(G)** Cell migration was assessed by wound healing assay (n = 5). Data was analyzed by one-way ANOVA test. **P < 0.01, ***P < 0.001. **(H)** Cell invasion was measured by Transwell assay (n = 4). Data was analyzed for statistical difference by one-way ANOVA test. ***P < 0.001. **(I)** Western blots of E-cadherin, N-cadherin, Snail and ALKBH5 in SK-OV3 cells transfected with empty vector (Vector), wild type ALKBH5 (ALKBH5-WT) and inactive mutant H204A ALKBH5 (ALKBH5-H204A) plasmid.

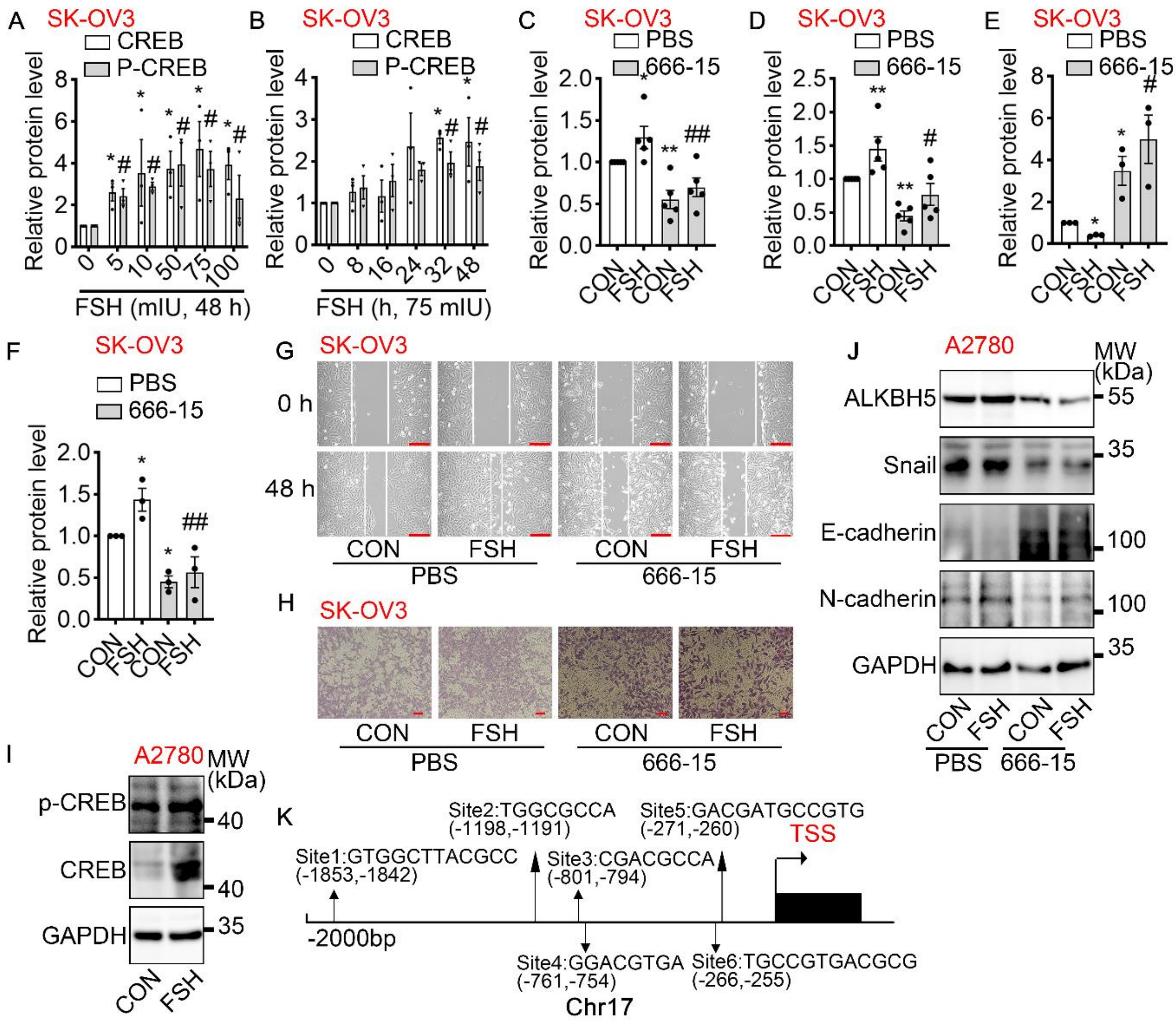

Supplementary figure 4. FSH activates CREB to elevate ALKBH5 expression. **(A, B)** Western blot quantification of p-CREB/CREB in SK-OV3 cells were treated with different concentrations of FSH for various time points (*n* = 3), data was analyzed by Mann-Whitney U test. **P* < 0.05 *versus* CON (CREB), #*P* < 0.05 *versus* CON (p-CREB). Western blot quantification of Snail **(C)**, ALKBH5 **(D)** E-cadherin **(E)** and N-cadherin **(F)** in SK-OV3 cells pretreated with CREB inhibitor, followed by treatment of FSH (*n* = 3 - 5), data was analyzed by Mann-Whitney U test. **P* < 0.05, ***P* < 0.01 *versus* CON group transfected with scramble siRNA, #*P* < 0.05, ##*P* < 0.01 *versus* FSH group transfected with scramble siRNA. **(G)** Representative images of wound healing in SK-OV3 cells pretreated with 666-15, followed by treatment of FSH (75 mIU, 48 h). Scale bar = 100 µm. **(H)** Representative images of Transwell invasion in SK-OV3 cells pretreated with 666-15, followed by treatment of FSH (75 mIU, 48 h) or PBS. Scale bar = 250 µm. **(I)** Western blots of p-CREB and CREB in A2780 cells with treatment of FSH. **(J)** Western blots of ALKBH5, Snail, E-cadherin and N-cadherin expression in A2780 cells pretreated with 666-15, followed by treatment of FSH (75 mIU, 48 h). **(K)** Schematic graph of the genomic locations of ALKBH5 and CREB potential binding sites in the promoter region of *ALKBH5* host gene chromosome 17.

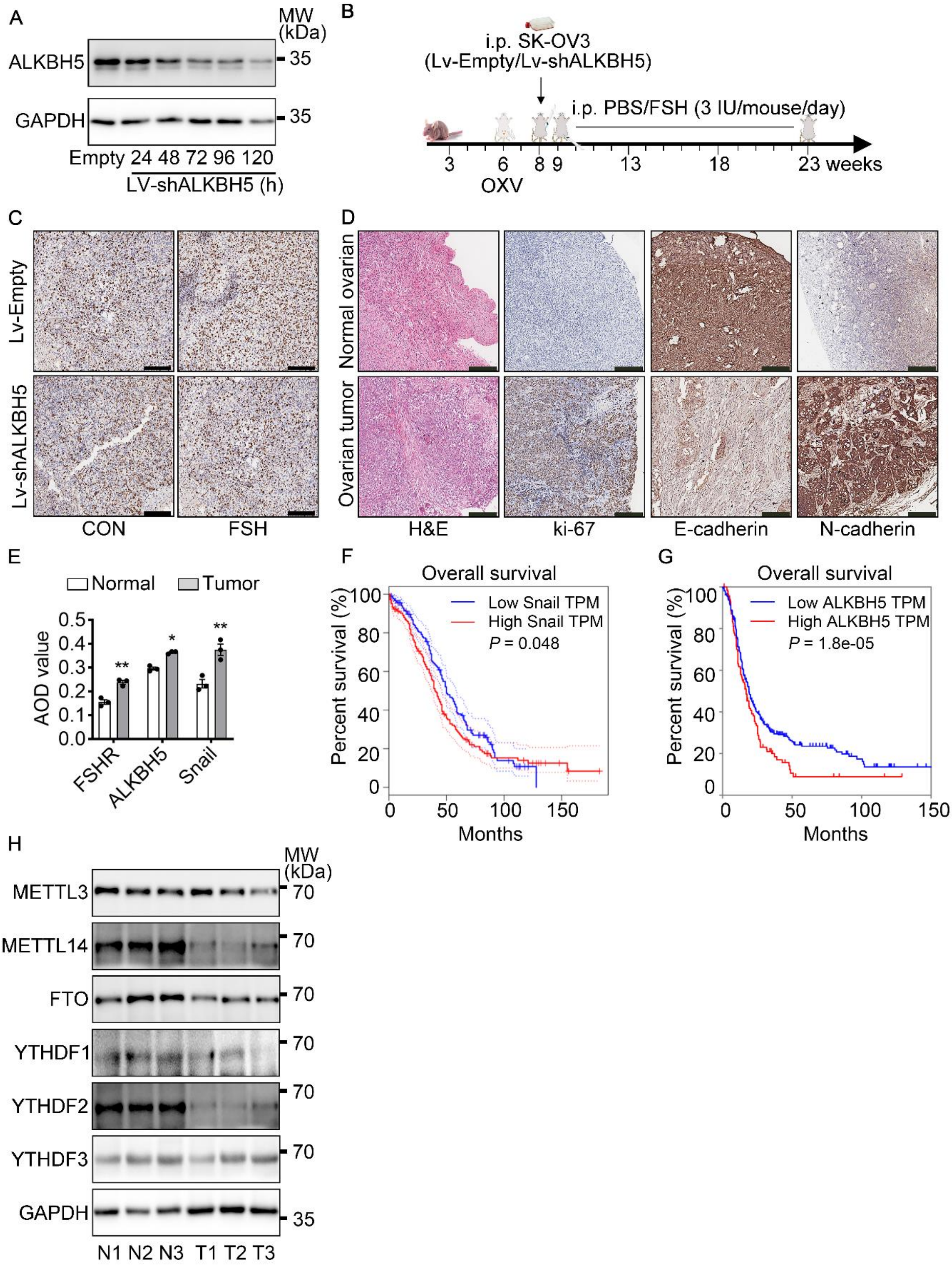

Supplementary figure 5. FSHR and Snail expression are positively associated with ALKBH5 expression. **(A)** Western blots of ALKBH5 in SK-OV3 cells transfected with a control (Lv-Empty) or shALKBH5 (Lv-shALKBH5) luciferase-tagged lentivirus. **(B)** Experimental timeline of bilateral ovariectomy (OVX) surgery, xerograph and FSH administration. **(C)** SK-OV3 xenografts were sectioned and stained for Ki-67 expression using IHC. Scale bar = 200 μm. **(D)** H&E, ki-67, E-cadherin and N-cadherin in tissue sections of normal and tumor ovarian tissues. Scale bar = 200 μm. **(E)** Histograms of average optical density (AOD) showed

the average immunoreactivity of FSHR, ALKBH5 and Snail in tissue sections of normal and tumor ovarian tissues ($n = 3$), data was analyzed by Student's t tests. *$P < 0.05$, **$P < 0.01$. **(F)** Survival analysis of ovarian cancer with high or low *Snail* mRNA using Gene Expression Profiling Interactive Analysis (GEPIA, http://gepia.cancer-pku.cn/index.html). **(G)** Kaplan-Meier analysis of overall survival in 614 ovarian cancer patients with various *ALKBH5* mRNA expression (https://kmplot.com/analysis/index.php?p=service). **(H)** Western blots of METTL3, METTL14, FTO and YTHDF1/2/3 protein in normal ovarian tissue (N) and ovarian tumors (T).
